# Plant-Derived Anti-Virulence Metabolites Targeting Gram-Negative Phytopathogens: Recent Advances and Perspectives

**DOI:** 10.3390/metabo16100768

**Published:** 2026-10-12

**Authors:** Ying Tang, Xuning Liu, Cuiyang Zhang, Binbin Huang, Ye Li, Zhaohui Guo, Qingshu Liu, Liyang Wu, Jie Du

**Affiliations:** 1 Institute of Microbiology, Hunan Academy of Agricultural Sciences, Changsha 410125, China; 2 Hunan Province Engineering Research Center for Agricultural Microbiology Application, Hunan Institute of Microbiology, Changsha 410009, China; 3 National Collection of Plant-Associated Microbes (Hunan), Changsha 410009, China; 4 Changsha Technology Innovation Center for Exploration and Utilization of Agricultural Microbial Germplasm Resources, Changsha 410009, China

**Keywords:** plant-derived metabolites, anti-virulence, gram-negative phytopathogenic bacteria, quorum sensing, type III secretion system, plant–pathogen interactions

## Abstract

Plant specialized metabolites play important roles in plant–microbe interactions. Beyond direct antimicrobial activity, some can attenuate pathogen virulence without markedly inhibiting microbial growth. Many gram-negative phytopathogenic bacteria cause important crop diseases and possess interconnected virulence systems, providing multiple opportunities for intervention by plant-derived anti-virulence metabolites. In this review, we summarize recent advances in these metabolites against gram-negative phytopathogenic bacteria according to three functional levels: virulence signal decision-making, virulence execution, and pathogenic ecological phenotypes. Particular attention is given to the strength of mechanistic evidence, including target engagement, pathway modulation, and disease-related outcomes. We further interpret these activities in the chemical-ecology context of plant–pathogen interactions and discuss the challenges and prospects of developing plant-derived metabolites as anti-virulence agents.

## 1. Introduction

Through long-term adaptation to environmental stresses and co-evolution with microorganisms, plants have evolved a rich repertoire of specialized metabolites. These metabolites not only participate in plant growth, development, and environmental adaptation but also constitute an important chemical basis for defense against pathogen infection. For a long time, attention focused mainly on their direct antimicrobial activities, leading to the discovery of numerous bactericidal natural products. However, a growing body of research indicates that a subset of plant specialized metabolites do not directly inhibit pathogen growth but instead contribute to plant defense by modulating pathogen virulence systems and interfering with the infection process [1]. Such metabolites illustrate the complex chemical communication between plants and pathogens and show how plant compounds can regulate pathogen behavior. This perspective may improve our understanding of plant innate immunity and guide the development of new disease-control strategies.

At present, chemical control of bacterial plant diseases still relies primarily on bactericides and antibiotics that directly inhibit or kill the pathogen. However, this strategy continuously drives the emergence of resistant populations, shortening the effective lifespan of agents, increasing control costs, and accumulating ecological risks. In recent years, the anti-virulence strategy originating from medical microbiology has gradually attracted attention in plant pathology [2,3,4,5]. Rather than eliminating the pathogen directly, this strategy weakens infectivity by interfering with the virulence mechanisms essential for pathogenesis, while reducing the selective pressure imposed by direct killing. It is therefore regarded as an important research direction for addressing the problem of drug resistance [6]. This review focuses on plant-derived anti-virulence compounds that attenuate defined pathogen-side processes contributing to infection or disease, at concentrations that have minimal effects on bacterial growth or viability under the conditions tested. Host-mediated disease resistance is therefore considered outside the main scope of this review, although it may also reduce disease severity. We distinguish evidence for target engagement, pathway modulation, and disease-related activity. Direct binding assays support physical target engagement, while target-site mutagenesis or rescue experiments can further establish target or pathway dependence. Changes in reporters or gene expression support pathway-level modulation, whereas changes in virulence-associated phenotypes indicate functional effects downstream of the pathway. Plant infection or disease assays provide functional evidence of disease-related activity but do not alone identify the molecular target. Accordingly, each compound is discussed according to the type and strength of evidence reported in the cited study.

Gram-negative phytopathogenic bacteria are among the most important causal groups of bacterial plant diseases, including *Pseudomonas*, *Xanthomonas*, *Ralstonia*, *Pectobacterium*, and *Dickeya*. Their infection processes generally depend on highly coordinated virulence regulatory networks, encompassing key processes such as quorum sensing (QS), the type III secretion system (T3SS), extracellular enzymes, biofilm formation, motility, and oxidative-stress adaptation. Many of these virulence-associated processes share conserved functional features across different pathogens and are coordinately regulated by key regulatory nodes, making them important potential targets for plant-derived anti-virulence metabolites [7,8].

In recent years, research on plant-derived anti-virulence metabolites has advanced considerably. Existing studies show that salicylic acid (SA) can interfere with quorum-sensing signaling [9], whereas erucamide targets a key structural component of the T3SS [10]. Other plant-derived phenolic acids, phenolic volatiles, and aromatic specialized metabolites have also been reported to modulate virulence-gene expression, biofilm formation, and motility at concentrations that do not markedly affect pathogen growth [11,12].

**Figure 1:**
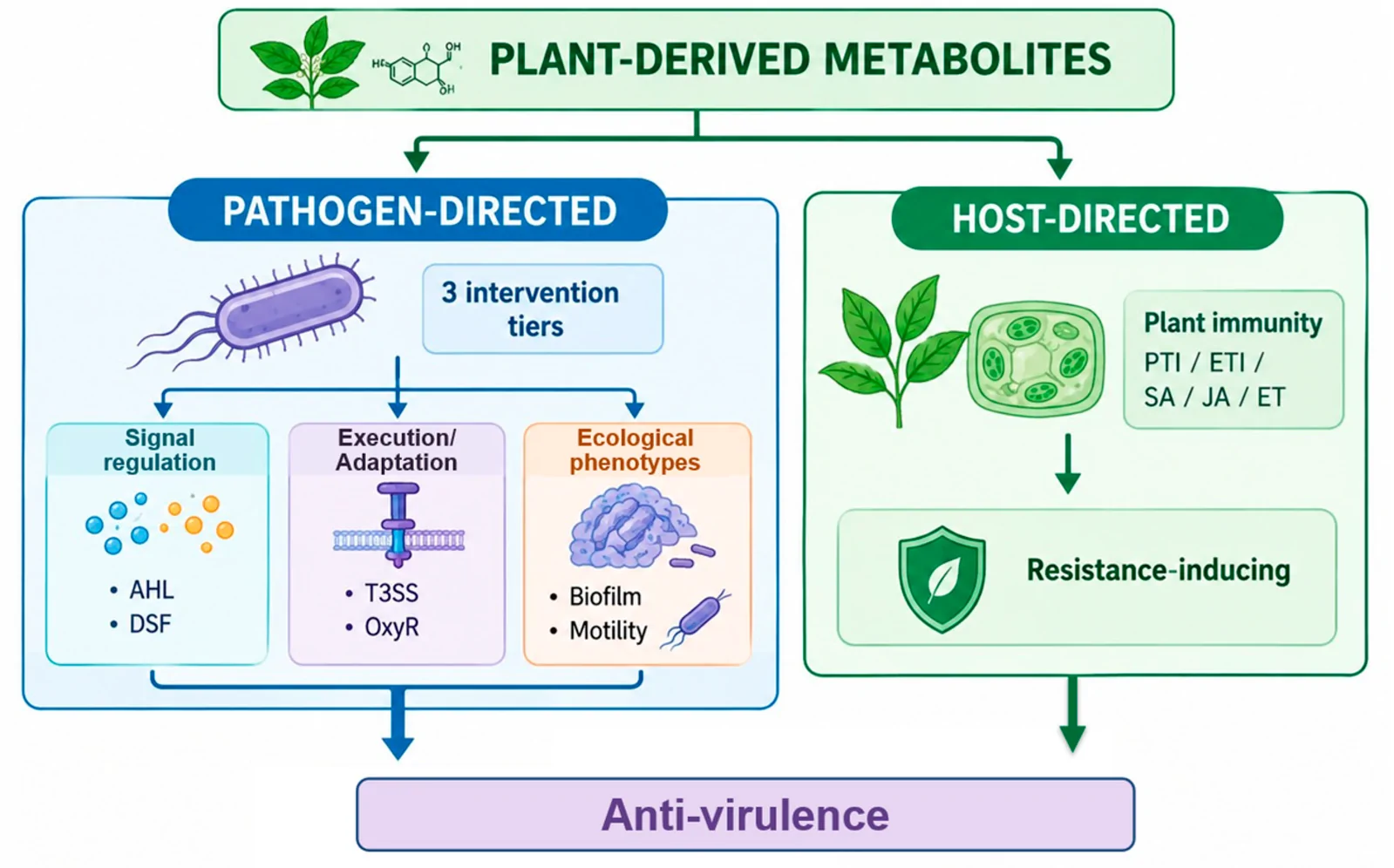
Framework distinguishing pathogen-directed anti-virulence activity from host-mediated induced resistance. Note: In this review, pathogen-directed anti-virulence activity refers to plant-derived compounds that have minimal effects on bacterial growth at the active concentration and interfere with defined pathogen-side virulence processes. The pathogen-side processes discussed in this review are organized into three levels: signal decision-making, including QS and DSF signaling; virulence execution, including the T3SS and secreted virulence factors; and pathogenic phenotypes, including biofilm formation and motility.

Research on plant-derived metabolites that regulate phytopathogen virulence has increased rapidly. However, existing reviews have focused mainly on particular pathogens, individual virulence systems, or specific mechanisms such as quorum sensing [4,13,14]; an integrated overview of their modes of action, common principles, and application potential across different virulence levels is still lacking. This review focuses on gram-negative phytopathogenic bacteria and classifies plant-derived anti-virulence metabolites into three functional levels: virulence signaling, execution, and ecological phenotypes (Figure 1). Within this framework, we summarize their mechanisms of action, representative targets, and functional features, and further discuss their ecological significance in plant–pathogen chemical communication and their prospects and challenges for sustainable disease control. Natural products that act mainly through direct antibacterial activity are included only where necessary for comparison [15,16,17].

## 2. Intervention at the Signal-Decision Level: Quorum Sensing and DSF

Quorum sensing (QS) is a key regulatory mechanism that enables gram-negative phytopathogens to coordinate virulence gene expression in a cell density-dependent manner, thereby helping determine when infection-related processes are initiated. Accordingly, targeting this “virulence switch” may reduce pathogenicity without necessarily causing direct growth inhibition or killing, making QS one of the earliest regulatory layers investigated in plant-derived anti-virulence research. Among plant pathogenic bacteria, the best-characterized QS systems include the LuxI/LuxR system, which uses acyl-homoserine lactones (AHLs) as signaling molecules, and the Rpf regulatory system centered on the diffusible signal factor (DSF) [18]. As upstream signaling systems in virulence regulation, both QS and DSF control a range of downstream population-level behaviors and virulence-associated processes. Plant-derived metabolites may therefore interfere with these pathways by affecting signal synthesis, perception, or response, or by mimicking endogenous signals, thereby altering pathogen behavior and virulence expression (Table 1).

### 2.1. Direct Interference with AHL Quorum Sensing: SA and Other Plant-Derived Phenolics

**Figure 2:**
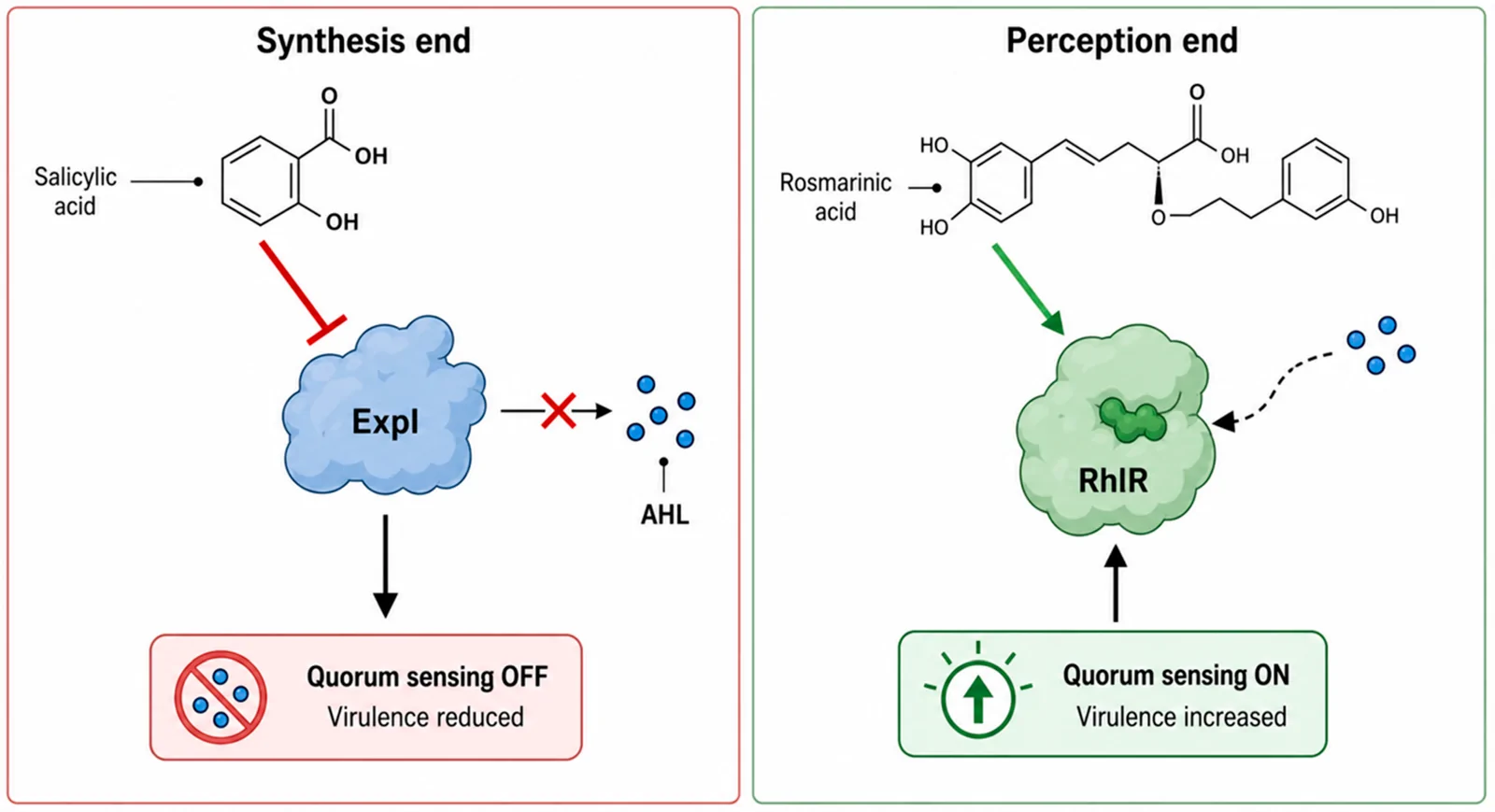
Contrasting action sites and functional outcomes of quorum-sensing modulation by plant-derived compounds: inhibition of signal synthesis versus receptor activation. Note: Salicylic acid inhibits AHL synthesis through ExpI, whereas rosmarinic acid activates RhlR and QS-dependent responses. Red blunt arrows, inhibition; red crossed arrows, blocked signal production; green arrows, activation; dashed arrows, native signal recognition.

Within AHL-type quorum sensing, the plant defense hormone SA is currently the plant-derived anti-virulence compound with the strongest mechanistic evidence. Using binding assays and genetic analyses, Joshi et al. found that SA competes for the SAM-binding site of the AHL synthase ExpI in the soft-rot pathogen *Pectobacterium*, thereby inhibiting AHL synthesis. At concentrations that do not affect pathogen growth, AHL levels decreased in a dose-dependent manner, and pathogenicity was reduced to a level comparable to that of the *expI* mutant, indicating that ExpI is a direct target through which SA exerts its anti-virulence activity [9] (Figure 2). Building on this finding, the SA-derived compound disalicylic acid exhibited stronger AHL-inhibitory activity and improved control of soft rot [23], suggesting that SA may serve as a promising lead scaffold for further structural optimization. Given the relatively high conservation of LuxI-type AHL synthases among diverse plant pathogens, anti-virulence strategies targeting this enzyme may have broader application potential.

Beyond SA, plant phenolics with diverse chemical scaffolds can also target the AHL quorum-sensing system. For example, carvacrol and eugenol have pathway-level and disease-related support for QS interference in *Pectobacterium*. They reduced AHL accumulation and QS-associated virulence outputs, and exogenous AHL restored virulence in plant infection assays [11]. Pun et al. further showed that phloretin, an apple-derived dihydrochalcone phytoalexin, reduces AHL production in the soft-rot pathogen *P. brasiliense* and attenuates several downstream virulence-associated outputs, including virulence-related gene expression, motility, biofilm formation, and plant infection. A heterologous ExpI assay further supported inhibition of ExpI-dependent AHL production at a growth-sparing concentration [19]. For all three compounds, proposed interactions with ExpI or ExpR are based on docking; direct binding and compound-specific target-mutant validation have not been reported.

Overall, the AHL system is an upstream regulator of pathogen virulence and is one of the most extensively studied signaling targets of plant-derived anti-virulence compounds. Current research has focused mainly on AHL-dependent soft-rot pathogens such as *Pectobacterium*. However, QS systems differ in composition and regulatory architecture among plant pathogens, and whether anti-virulence effects mediated through AHL signaling are broadly applicable across pathogens remains to be determined. This limitation is further illustrated by the counterexample of rosmarinic acid discussed below.

### 2.2. A Critical Constraint on the Direction of Action: Rosmarinic Acid Activates Quorum Sensing

The interaction between a plant-derived compound and a QS system does not necessarily attenuate virulence; the outcome depends on the molecular target, regulatory mode, and biological context. Unlike SA, which inhibits AHL signal synthesis, Corral-Lugo et al. found that rosmarinic acid can directly bind the QS receptor RhlR in *P. aeruginosa* and act as a natural HSL mimic to activate its transcriptional regulatory activity.

At concentrations that do not affect bacterial growth, rosmarinic acid induced QS-dependent gene expression and promoted pyocyanin and elastase production as well as biofilm formation, resulting in enhanced virulence-associated phenotypes [20] (Figure 2). The response was concentration-dependent, with enhanced RhlR-mediated reporter activity at 0.5–5 mM under the conditions tested. Although the authors proposed premature QS activation as a possible plant defense, the study did not test whether it reduces infection success, pathogen fitness, or disease severity under plant-associated conditions. Because *P. aeruginosa* is an opportunistic pathogen with a broad host range rather than a typical phytopathogenic bacterium, this study is discussed mainly as a mechanistic reference. It highlights the context-dependent and bidirectional effects of plant-derived QS modulation.

### 2.3. The DSF-Rpf System: A Promising but Underexploited Signaling Target

The diffusible signal factor (DSF) family comprises cis-2-unsaturated fatty acids that regulate virulence-associated traits in several plant-pathogenic bacteria, particularly *Xanthomonas* spp. In *Xanthomonas*, DSF is synthesized by RpfF and perceived by the membrane-associated sensor kinase RpfC, which transduces the signal to the response regulator RpfG. Through its HD-GYP domain, RpfG modulates intracellular cyclic-di-GMP levels, thereby linking DSF perception to extracellular enzyme secretion, biofilm formation, motility, and other pathogenicity-associated processes [18,24]. Because the DSF-Rpf circuit coordinates multiple virulence-related traits and is relatively conserved among *Xanthomonas* species, it has been proposed as an anti-virulence target [25]. Although endogenous DSF has been shown to bind directly to RpfC and activate its kinase activity, comparable evidence that plant-derived compounds directly inhibit core DSF-Rpf signaling components such as RpfC or RpfG is still lacking [26,27].

Compared with the AHL system, the DSF-Rpf pathway has been examined less often as a target of plant-derived anti-virulence compounds, and the available evidence mainly concerns changes in DSF-family signal abundance or downstream phenotypes. The clearest example is salicylic acid (SA), although its effect is not consistent with a simple anti-virulence outcome. In *Xanthomonas campestris* pv. *campestris* (Xcc), SA promotes RpfB-dependent turnover of DSF and BDSF under acidic, plant-associated conditions, apparently through changes in culture and cytoplasmic pH rather than direct activation of RpfB or direct inhibition of the RpfC-RpfG signaling module. Despite reducing DSF-family signal levels, SA-treated Xcc showed enhanced virulence in cabbage, indicating that reduced signal abundance does not necessarily translate into disease suppression [28]. This complexity is further underscored by evidence that the DSF signal itself can interfere with plant immunity, including suppression of flagellin-triggered immune responses in *Arabidopsis* through effects on sterol biosynthesis [29]. By contrast, thymol and citronellal have been reported to attenuate DSF-associated phenotypes in *X. oryzae* pv. *oryzae*, including reduced biofilm formation, extracellular enzyme production, and motility. However, their proposed interaction with RpfF is based mainly on molecular docking and phenotypic observations, and direct binding or target-engagement evidence is still lacking [21,22]. These observations highlight the difficulty of developing DSF-pathway inhibitors: RpfC is a membrane-associated multi-domain sensor, RpfG is embedded in the broader cyclic-di-GMP network, and DSF-family signal composition varies across species and host-associated conditions, as illustrated by the predominance of BDSF during cabbage infection by Xcc [30]. Therefore, changes in signal abundance, gene expression, biofilm formation, or docking predictions should be interpreted cautiously unless supported by direct target-engagement evidence, genetic validation, and whole-plant infection assays.

Related fatty-acid signaling occurs in *R. solanacearum*, but through the distinct Phc system: PhcB produces 3-OH-PAME or 3-OH-MAME, which are perceived by PhcS–PhcR and regulate the master virulence regulator PhcA [18,31]. Although synthetic antivirulence molecules targeting the Phc quorum-sensing system of *R. solanacearum* have been developed [32], experimentally validated plant-derived compounds that directly target this fatty-acid quorum-sensing system remain scarce. Recent evidence indicates that PABA can suppress multiple quorum-sensing outputs, including 3-OH MAME production, but its primary validated target is the anthranilic acid regulator RaaR rather than a core Phc component [33].

## 3. Blocking Virulence Output Through the Type III Secretion System

The type III secretion system (T3SS) is a key secretion apparatus through which gram-negative phytopathogenic bacteria deliver effector proteins into host cells and interfere with plant immunity; it is also one of the most extensively studied targets at the virulence-execution level for plant-derived anti-virulence compounds.

The T3SS of plant-pathogenic bacteria is encoded mainly within the *hrp/hrc* gene cluster, which contains conserved *hrc* genes encoding core components of the injectisome. Among these, HrcC is the outer-membrane secretin, whereas HrcN is the ATPase that provides energy for T3SS function. By contrast, the upstream transcriptional regulatory networks show substantial variation among pathogens. For example, *Xanthomonas* species are predominantly governed by the HrpG–HrpX (HrpB) cascade, whereas *D. dadantii* is regulated mainly by the alternative sigma factor HrpL and its upstream regulators HrpR/HrpS and RpoN [34]. This combination of structural conservation and regulatory divergence has important implications for the breadth of different anti-virulence strategies. Targeting conserved structural components may provide broader pathogen coverage, whereas compounds acting on transcriptional regulators or upstream signal-perception pathways are more likely to show pathogen-specific activity.

Based on their level of action, plant-derived T3SS-targeting compounds can be broadly divided into two categories. One group directly interferes with injectisome assembly and prevents formation of a functional secretion apparatus. The other acts on transcriptional regulation of the *hrp/hrc* gene cluster or upstream signal-perception pathways, thereby reducing T3SS-related gene expression. In addition, pathogens must maintain infection through adaptive processes such as antioxidant defense; targeting these processes may likewise impair pathogenicity. Representative plant-derived anti-virulence molecules acting at these different levels are discussed below (Table 2).

### 3.1. Direct Targeting of T3SS Structural Components

Among plant-derived T3SS inhibitors, erucamide is currently one of the few representative plant-derived anti-virulence compounds supported by a well-defined molecular target, mechanistic evidence, and genetic validation. In their investigation of plant innate immunity, Miao et al. identified erucamide among the metabolites induced by PAMPs. Erucamide is a fatty acid amide whose accumulation depends on the immune co-receptor BAK1 and has been detected across diverse plant species, including *Arabidopsis*, rice, wheat, maize, and tomato [10].

Mechanistic studies show that erucamide does not affect the expression of T3SS-related genes but instead binds directly to the T3SS outer-membrane secretin HrcC, blocking injectisome assembly and thereby inhibiting effector secretion. At concentrations that do not affect pathogen growth, erucamide markedly reduced effector secretion, indicating that its anti-virulence effect does not result from direct antibacterial activity. Genetic analysis further confirmed that mutation of a key HrcC binding site abolished both erucamide binding and its inhibitory activity, establishing HrcC as its direct molecular target. Moreover, experimentally manipulating erucamide levels *in planta* altered plant resistance to T3SS-dependent pathogens, further supporting its biological role.

As a conserved T3SS structural component, HrcC represents a promising target for the development of anti-virulence strategies with potentially broad pathogen coverage; this concept has also been highlighted in related commentary [41]. In addition, structure–activity relationship studies indicate that the amide group and long-chain fatty-acid moiety are important for maintaining activity, providing a basis for subsequent lead optimization.

### 3.2. Indirect Modulation of T3SS Through Transcriptional Regulation and Signal-Perception Networks

Unlike erucamide, which directly interferes with T3SS structural assembly, most plant-derived T3SS inhibitors do not directly target the secretion apparatus itself but instead interfere with upstream regulatory processes, including hrp/hrc gene-cluster expression and host-signal perception, thereby reducing T3SS formation and effector-delivery capacity. This mode of action has been investigated most extensively in *P. syringae*.

Plant phenolic compounds are currently among the best-characterized classes of T3SS-regulating molecules. Kang et al. identified 4-hydroxybenzoic acid and vanillic acid from *Sedum middendorffianum* and demonstrated in *P. syringae* pv. *tomato* DC3000 (Pst DC3000) that both compounds markedly suppressed T3SS-related gene expression and reduced pathogenicity without affecting bacterial growth [35]. The same group further identified the resveratrol oligomers hopeaphenol, isohopeaphenol, and ampelopsin A from root extracts of grapevine (*Vitis vinifera*) and found that these compounds down-regulated the transcription of key T3SS-associated genes, including *hrpA*, *hrpL*, and the effector gene *hopP1* [36]. These studies support growth-sparing T3SS pathway modulation and disease-related activity, but neither demonstrated direct binding of the compounds to a T3SS component or upstream regulatory protein.

Beyond directly affecting virulence-gene expression, some plant polyphenols can act on upstream T3SS signal-transduction systems. Xie et al. found that plant polyphenols, including tannic acid, 1,2,3,4,6-pentagalloyl glucose (PGG), and epigallocatechin gallate (EGCG), target the RhpRS two-component system in *P. syringae* pv. *phaseolicola*. By interacting with the sensor histidine kinase RhpS, these compounds inhibit its phosphatase activity, thereby increasing the phosphorylation level of RhpR and altering downstream regulatory networks to suppress T3SS activation [37].

Similar anti-virulence strategies have gradually extended to other important plant pathogens. In *Dickeya* spp., Hu et al. identified multiple plant-derived T3SS inhibitors, including SA, p-hydroxybenzoic acid, cinnamyl alcohol, p-coumaric acid, and hydrocinnamic acid. These compounds downregulated T3SS-related gene expression, particularly that of the master regulator gene *hrpL*, and weakened the tobacco hypersensitive response; these anti-virulence effects were associated with partial control of several *Dickeya*-associated diseases without affecting bacterial growth [42]. For *R. solanacearum*, Guo et al. reported the mandelic-acid derivative F-24 as a T3SS-modulating compound. F-24 reduced the expression of *hrpY* and multiple effector genes and down-regulated the HrpB regulon, resulting in an approximately 50% reduction in tomato bacterial-wilt incidence without substantially reducing bacterial populations in planta [43]. Although F-24 is a semi-synthetic derivative rather than a naturally occurring plant metabolite, it provides an illustrative example of how plant-derived scaffolds can be further optimized for anti-virulence activity. In *X. oryzae* pv. *oryzae*, the phenolic acid *o*-coumaric acid (OCA) specifically inhibited *hrp*/*hrc* gene-cluster expression without affecting growth-associated genes, including flagellar biosynthesis genes [39]. Cinnamic-acid-type molecules such as CZ-1, CZ-4, and CZ-9, identified through activity-guided screening of natural phenolic derivatives, likewise down-regulated T3SS expression via the HrpG-HrpX cascade and reduced rice lesion development at concentrations that did not affect bacterial growth [44].

However, compared with strategies that directly target conserved structural components, plant-derived compounds acting on transcriptional regulation and signal-perception networks show greater pathogen dependence, and their effects are not always consistent. Li et al. found that the plant cell-wall-derived phenolic acid *p*-coumaric acid (PCA) suppresses T3SS gene expression in *D. dadantii* through the HrpX/HrpY two-component system and the Rsm network, while its hydroxamic-acid derivative TS103 exhibits approximately eightfold greater inhibitory activity than PCA [38,45]. In contrast, the structural analogs OCA and *trans*-cinnamic acid (TCA) induce T3SS expression in the same strain through the *rsmB*-RsmA pathway [46]. OCA also illustrates pathogen-dependent activity, as it induces T3SS expression in *Dickeya* [46], but suppresses T3SS expression in *X. oryzae* pv. *oryzae* [39]. In addition, SA, eugenol, chlorogenic acid, resveratrol, and cinnamic acid suppress T3SS expression in some pathogens but may instead induce T3SS expression and enhance virulence in *R. solanacearum* [4]. These observations indicate that the anti-virulence activity of plant-derived compounds cannot be predicted solely from their structural similarity; the composition and regulatory logic of the pathogen’s signaling network are critical determinants of the direction of action.

Therefore, plant-derived strategies based on transcriptional regulation or signal-perception systems, such as RhpRS, HrpX/HrpY, and Rsm, are often constrained by pathogen-specific regulatory networks. This contrasts with erucamide, which targets the relatively conserved T3SS structural component HrcC.

### 3.3. Interfering with Oxidative-Stress Adaptation: Sulforaphane Targets Pathogen Antioxidant Defense

Distinct from strategies targeting the T3SS secretion apparatus, another class of plant-derived anti-virulence molecules acts by weakening the pathogen’s ability to withstand host defense. The reactive oxygen species (ROS) burst generated during early infection is an important immune barrier. Gram-negative pathogens counter this stress through OxyR-regulated antioxidant systems, including the catalases KatA and KatG, which detoxify ROS and support survival and colonization. Disrupting this oxidative-stress adaptation can therefore attenuate pathogenicity without directly inhibiting bacterial growth.

Sulforaphane (SFN) is an isothiocyanate defense metabolite produced by cruciferous plants. Wang et al. found that SFN directly binds the oxidative-stress regulator OxyR in Xcc, suppressing the expression of antioxidant enzyme genes such as *katA* and *katG* and thereby compromising the pathogen’s ability to tolerate ROS, including H_2_O_2_. This interference with antioxidant defense weakens the pathogen’s capacity to withstand the oxidative burst associated with plant immunity. At concentrations that do not affect bacterial growth, SFN markedly reduces pathogen virulence, indicating that its anti-virulence activity is primarily attributable to disruption of oxidative-stress adaptation rather than direct antibacterial action. Given the high conservation of OxyR among gram-negative pathogens, this strategy may have potential for broader-spectrum application [40].

However, the anti-virulence activity of SFN is strongly species-dependent. In Xcc, which carries the *sax* gene cluster and can detoxify SFN, bacterial growth is inhibited only at relatively high SFN concentrations, whereas anti-virulence effects can occur at substantially lower concentrations, providing a clear anti-virulence window. By contrast, in other *Xanthomonas* species lacking this detoxification system, SFN causes growth inhibition at lower concentrations, potentially overlapping with those required for anti-virulence effects and thereby narrowing the anti-virulence window. Thus, pathogen-intrinsic detoxification capacity can influence whether SFN exhibits a distinct anti-virulence window or predominantly antibacterial activity [47].

This phenomenon reflects a chemical arms race driven by long-term plant–pathogen co-evolution. Many plant pathogens have evolved detoxification mechanisms exemplified by the *sax* gene cluster and the SaxA isothiocyanate hydrolase, which can degrade, export, or otherwise detoxify isothiocyanates, thereby weakening plant chemical defenses [48,49,50]. Therefore, beyond target conservation, the application potential of plant-derived anti-virulence compounds should also be evaluated in light of pathogen detoxification capacity and potential adaptive responses. This represents an important consideration for the further development of isothiocyanate-based compounds.

Beyond the T3SS, extracellular enzymes and toxins are important virulence factors, particularly in soft-rot pathogens *Pectobacterium* and *Dickeya*, where pectinases and cellulases contribute directly to tissue maceration. However, plant-derived compounds that directly target these factors while minimally affecting bacterial growth remain scarce, with most reported effects on extracellular enzyme production mediated indirectly through upstream systems such as quorum sensing (see Section 2.1). Direct targeting of extracellular enzyme secretion, maturation, or activity therefore represents an underexplored avenue for execution-level anti-virulence strategies.

## 4. Regulation of Pathogen Ecological-Adaptation Phenotypes: Biofilm and Motility

Biofilm formation and flagellar motility are important ecological phenotypes that enable plant pathogens to colonize, disseminate, and adapt to their environments. These traits are also closely associated with pathogen persistence and disease development, making them relevant downstream phenotypes for evaluating anti-virulence activity. Several plant-derived compounds have been reported to modulate these traits. SA, carvacrol, and eugenol inhibit biofilm formation in *Pectobacterium*, whereas phloretin affects motility and fitness in *Pectobacterium brasiliense* [11,19]. In *Xanthomonas* and *Ralstonia*, biofilm formation and motility are also important phenotypic outputs associated with the DSF-Rpf regulatory system [24,25].

Because biofilm formation and motility are regulated by interconnected pathways, changes in these phenotypes are useful readouts of pathogen behavior but do not by themselves demonstrate reduced pathogenicity [51]. Their relevance to anti-virulence activity should therefore be evaluated together with pathway-level evidence and infection or disease assays.

## 5. Chemical-Ecology Context of Plant-Derived Anti-Virulence Compounds

From a chemical-ecology perspective, plant-derived anti-virulence compounds can be viewed as chemical mediators that alter pathogen behavior rather than simply inhibiting pathogen growth. These chemical interactions can be organized using a three-tier intervention model, in which these compounds act at different levels of the pathogen’s signal-decision process, virulence execution machinery, and infection-associated phenotypes. This framework also emphasizes that these effects are not uniformly suppressive and depend on compound structure, molecular target, concentration, and ecological context. Closely related metabolites may differ in activity, and a single compound can affect multiple targets or produce different outcomes in different organisms [52].

This context dependence is especially important when interpreting plant metabolites that affect pathogen regulatory systems. Some metabolites may interfere with virulence regulation in ways that are potentially beneficial to the host, whereas others may be exploited by pathogens as host-derived cues. For example, rosmarinic acid was reported to act as an HSL mimic that directly binds the QS regulator RhlR and activates RhlR-mediated transcription in *P. aeruginosa* [20]. Although this was proposed to trigger premature quorum sensing as a plant defense strategy, its ecological role during infection remains to be experimentally demonstrated. Conversely, putrescine functions as a host-derived signal perceived by *P. syringae* pv. *actinidiae* through the histidine kinase BvgS, leading to T3SS activation and enhanced virulence during kiwifruit infection [53]. These examples show that plant-derived compounds can redirect pathogen decision-making, but the outcome may range from virulence attenuation to virulence promotion depending on the pathogen, target pathway, and infection context.

The multifunctionality of plant specialized metabolites further complicates their classification as anti-virulence compounds. Molecules originally characterized as phytoalexins, phytoanticipins, hormones, or defense signals may also affect quorum sensing, virulence-gene expression, secretion-system activity, biofilm formation, or host colonization [54,55]. For instance, SA is primarily known as a plant immune signal but can also alter DSF-family signal turnover in *Xanthomonas*, whereas erucamide has been described as an inducible defense metabolite that suppresses T3SS-associated virulence. Thus, labels such as “defense compound,” “signaling molecule,” and “anti-virulence factor” should be treated as context-dependent rather than mutually exclusive categories. Establishing the ecological significance of a candidate compound therefore requires linking molecular target engagement to pathogen behavior and disease outcome under host-associated conditions.

Spatial and temporal context helps determine whether a plant metabolite can reach a pathogen target at a biologically relevant concentration. Metabolites released into the apoplast may be accessible to extracellular pathogens, whereas compounds retained within plant cells may have limited access unless they are transported, released, or encountered during tissue invasion. Intracellular pathogens, in turn, may encounter metabolites in different compartments. Local concentration, secretion, transport, chemical stability, and microbial transformation can therefore influence whether an interaction observed in vitro also occurs during infection. Spatial metabolomics and mass spectrometry imaging (MSI) can map metabolite distributions at plant–pathogen interfaces and help assess whether these distributions are consistent with proposed mechanisms [56].

## 6. Mechanistic Boundaries and Application Challenges

Despite these emerging opportunities, several conceptual and practical barriers still limit the translation of plant-derived anti-virulence compounds from mechanistic discovery to field application. These challenges include functional classification, resistance evolution, ecological safety, and industrial development. Importantly, disease reduction does not by itself establish an anti-virulence mechanism, nor does plant origin necessarily imply safety. Their evaluation should therefore be based on clearly defined targets, molecular mechanisms, effective concentration ranges, and ecological effects.

### 6.1. The Boundary Between Anti-Virulence and Induced Resistance, and the Risk of Resistance

Some plant-derived compounds reduce disease through host-mediated defense activation rather than direct interference with pathogen virulence. Naringenin illustrates this distinction. In *Arabidopsis*, pretreatment with naringenin reduced disease symptoms and the population of Pst DC3000, although no direct antibacterial activity against the pathogen was detected in the in vitro assay. Naringenin treatment increased salicylic acid accumulation, promoted NPR1 monomerization and nuclear translocation, and induced pathogenesis-related gene expression. The protective effect was impaired in *npr1*, *mpk3*, and *mpk6* mutants, supporting a host-mediated resistance mechanism involving NPR1 and MAPK signaling. Naringenin is therefore best described here as an inducer of host resistance, rather than as a pathogen-directed anti-virulence metabolite [57].

Anti-virulence strategies are often considered less likely to impose strong resistance selection because they do not directly inhibit pathogen growth; however, weaker selection pressure does not mean that pathogens are unable to adapt [58]. Taking quorum sensing as an example, although quorum sensing itself is generally not essential for survival, its disruption can alter cooperation and competition within bacterial populations and thereby generate new selective pressures. Mellbye and Schuster demonstrated in *P. aeruginosa* that prolonged intervention against virulence-associated traits can likewise promote adaptive mutations [59]. The likelihood and route of adaptation may further depend on the nature of the molecular target. Compounds acting on complex regulatory networks may allow adaptation through signal rewiring or compensatory pathways, whereas compounds targeting highly conserved structural components, such as erucamide acting on T3SS HrcC assembly, may theoretically impose a higher adaptive barrier. Nevertheless, even such targets may remain susceptible to adaptation through target-site mutations or alternative pathways. Long-term experimental evolution and field-based studies are therefore needed to determine whether these theoretical differences in adaptive potential translate into meaningful differences in resistance risk.

### 6.2. “Plant-Derived” Is Not Inherently Safe: Ecological Risks and the Need for Rigorous Evaluation

Plant origin does not inherently imply ecological safety. Many plant-derived molecules exhibit broad biological activities; for example, essential-oil components and some phenolic compounds can affect diverse microorganisms and non-target organisms at higher concentrations [60]. Their field application therefore requires careful consideration of potential effects on rhizosphere and phyllosphere microbial communities and beneficial organisms. Moreover, the ecological effects of natural products are highly dependent on dose, exposure conditions, and environmental context, such that the same compound may exert stimulatory, inhibitory, or even bidirectional effects under different conditions. Therefore, the potential advantages of plant-derived anti-virulence compounds should not be attributed simply to their “natural” origin. They should be supported by evidence for a defined mechanism of action, an effective concentration range, environmental fate, and ecological safety.

### 6.3. Industrialization Bottlenecks from Compound Discovery to Field Application

The translation of promising plant-derived anti-virulence metabolites into practical products remains constrained by several interconnected bottlenecks. First, compound availability can limit development, as many active molecules occur at low levels in plants or exhibit inducible and tissue-specific accumulation, making a stable and economical supply difficult to achieve [61]. Structurally complex natural products may therefore face greater production and standardization challenges than relatively simple compounds with well-defined chemical sources, such as SA and erucamide. Second, variability in plant sources, extraction procedures, and metabolite composition can result in batch-to-batch differences in activity, emphasizing the need to identify the true active constituents and establish robust quality-control criteria. Third, laboratory efficacy does not necessarily translate into field performance. Ultraviolet radiation, temperature and humidity fluctuations, rain wash-off, and microbial degradation may reduce compound persistence and effective exposure, particularly for volatile compounds such as carvacrol and eugenol [62].

Recent field studies illustrate both the feasibility and the complexity of this translation. Yuan et al. demonstrated that the plant phenolic acid derivative TS108, a T3SS inhibitor targeting the CsrB–HrpS–HrpL regulatory cascade in *Erwinia amylovora*, could reduce fire blight under field conditions. Importantly, TS108 did not affect bacterial growth, supporting its classification as a virulence-targeted compound rather than a conventional bactericide. Across five independent field experiments conducted in four U.S. states between 2015 and 2022, TS108 treatment reduced blossom blight incidence by up to 96.7% relative to untreated trees. Importantly, TS108 did not inhibit T3SS expression in *D. dadantii* or *P. aeruginosa*, suggesting that its activity may depend on pathogen-specific regulatory architecture [63]. Recent work with essential-oil emulsions against *P. syringae* pv. *actinidiae* further illustrates the importance of formulation in field translation. Encapsulation improved the bioavailability and stability of the essential oils and enabled disease suppression on kiwifruit under both in vivo and field conditions. However, these formulations retained direct antibacterial activity in addition to effects on biofilm formation and T3SS induction [64]. Thus, field-level disease reduction should not automatically be interpreted as evidence of anti-virulence activity, particularly when antimicrobial and virulence-modulating effects coexist.

## 7. Conclusions and Future Perspectives

Plant-derived anti-virulence metabolites represent a complementary approach to conventional antimicrobial strategies by targeting pathogen behavior rather than relying primarily on growth inhibition. This review highlights three interconnected levels through which plant metabolites may influence bacterial pathogenicity. Importantly, these levels should not be interpreted as independent categories. Perturbation of an upstream regulatory component may propagate to multiple downstream phenotypes, while a change in a virulence-associated phenotype does not by itself establish an anti-virulence mechanism.

Several research gaps currently limit the development of this field. First, many reported activities are still supported mainly by transcriptional, reporter-based, phenotypic, or docking evidence, with limited direct biochemical or genetic validation of the proposed targets. Second, even when a putative target or pathway has been implicated, the causal chain linking target engagement to disease suppression, reduced virulence, and ultimately disease control remains insufficiently established for many candidates. Third, cross-pathogen efficacy cannot be assumed even when compounds target broadly conserved virulence systems. Pathogen-specific regulatory architecture, target variation, detoxification mechanisms, and infection biology may substantially influence susceptibility. Long-term experimental evolution and field monitoring are also needed to determine whether anti-virulence interventions can select for adaptive responses, target modification, signal rewiring, or metabolic detoxification. Finally, effects on beneficial and commensal microbiota remain insufficiently characterized, particularly when compounds influence regulatory pathways shared among multiple members of plant-associated microbial communities.

Future research should focus on establishing a complete causal chain from metabolite exposure to molecular target engagement, pathogen behavioral changes, and ultimately disease outcomes. Spatial and temporal information will be particularly important for determining whether the concentration and localization of a candidate metabolite are sufficient to reach its proposed pathogen target in planta. Such ecological information should be integrated with direct biochemical or ligand–protein interaction assays, structural analysis, and genetic validation to distinguish primary molecular targets from downstream network effects. At the same time, structure–activity relationship studies and comparative analyses across pathogens may help determine whether particular chemical features are associated with specific regulatory targets or pathogen susceptibilities. Machine-learning approaches could further integrate experimentally validated chemical–target–phenotype relationships with pathogen genomic and metabolomic data to identify previously unrecognized associations and prioritize candidates for experimental validation. Ultimately, the field should move from demonstrating isolated anti-virulence phenotypes toward establishing reproducible links between chemical structure, molecular mechanism, ecological context, and disease outcome.

## Figures and Tables

**Table 1:** Representative plant-derived anti-virulence metabolites and their targets, activity spectra, and validation levels (Signal-decision level).

Compound and Source	Target Pathogen and Spectrum	Intervention Level and Target	Validation Evidence
SA [9] Source: Widely distributed in plants	*Pectobacterium*, *Erwinia*, and other AHL-dependent pathogens	Signal decision: directly binds AHL synthase ExpI and inhibits signal synthesis	Direct binding + mechanistic + in vitro and in vivo validation
Carvacrol/eugenol [11] Source: Aromatic plant essential oils	*Pectobacterium*	Signal decision: inhibits ExpI/ExpR activity and reduces AHL levels	Molecular docking + in vitro activity + tuber disease control
Phloretin [19] Source: Apple; phytoalexin	*Pectobacterium brasiliense*	Signal decision: interferes with quorum sensing and reduces virulence and fitness	Single-pathogen phenotypic evidence
Rosmarinic acid [20] Source: Lamiaceae plants	*Pseudomonas aeruginosa* (counterexample)	Signal decision: binds RhlR as an agonist, thereby enhancing virulence	Direct binding + mechanistic evidence
Thymol [21] Source: *Thymus vulgaris*	*Xanthomonas oryzae* pv. *oryzae*	Signal: reduces DSF/BDSF-associated phenotypes and *rpfF* expression; RpfF interaction proposed by docking	Phenotypic + transcriptional evidence; target suggested by docking.
Citronellal [22] Source: *Citrus hystrix*	*Xanthomonas oryzae* pv. *oryzae*	Signal: Reduces biofilm formation, motility, virulence-related transcripts, and *rpfF* expression.	Phenotypic + transcriptional + leaf-infection evidence; direct target not shown

**Table 2:** Representative plant-derived anti-virulence metabolites and their targets, activity spectra, and validation levels (Execution level).

Compound and Source	Target Pathogen and Spectrum	Intervention Level and Target	Validation Evidence
Erucamide [10] Source: Various dicots and monocots	Multiple gram-negative phytopathogens	Execution: binds HrcC and blocks T3SS needle assembly	Direct binding + mechanistic + multi-pathogen + in vitro and in vivo validation
4-Hydroxybenzoic acid/vanillic acid [35] Source: *Sedum* spp.	Pst DC3000	Execution: suppresses *hrp* transcription	Reporter/transcript evidence + tomato disease assay; direct binding not demonstrated
Resveratrol oligomers [36] Source: Grape (*Vitis vinifera*)	Pst DC3000	Execution: downregulates *hrpA*, *hrpL*, and *hopP1*	Reporter/transcript evidence + tomato disease assay; direct binding not demonstrated
Polyphenols sensed by RhpRS [37] Source: Various plants	*Pseudomonas syringae* pv. phaseolicola	Signal regulation: polyphenol interaction with RhpS alters RhpRS signaling	RhpS interaction + Pro40 mutagenesis + in vitro activity and plant virulence assays
p-Coumaric acid [38] Source: a plant-derived phenolic acid	Multiple *Dickeya* species	Execution: suppresses T3SS through the HrpX/HrpY two-component system	Plant disease + pathway/regulatory evidence; direct binding not demonstrated
o-Coumaric acid [39] Source: a plant-derived phenolic acid	*Xanthomonas oryzae* pv. *oryzae*	Execution: suppresses *hrp/hrc* gene-cluster expression	Transcriptional/pathway evidence; direct binding not demonstrated
Sulforaphane [40] Source: Cruciferous plants (*Brassica* spp.)	*Xanthomonas* spp. tested; growth-sparing window varies by species	Execution: binds OxyR and suppresses oxidative-stress adaptation and virulence	MST binding + EMSA + OxyR and sax genetic evidence + virulence assays

Validation labels summarize the evidence reported in the source table. “Direct binding” is used only where binding evidence was reported; absence of demonstrated binding is stated explicitly where noted.

## Data Availability

No new data were created or analyzed in this study. Data sharing is not applicable to this article.

## Abbreviations

The following abbreviations are used in this manuscript:

QS	Quorum sensing
T3SS	Type III secretion system
SA	Salicylic acid
PTI	Pattern-triggered immunity
ETI	Effector-triggered immunity
JA	Jasmonic acid
ET	Ethylene
AHL	Acyl-homoserine lactones
DSF	Diffusible signal factor
Pst DC3000	*Pseudomonas syringae* pv. tomato DC3000
PGG	1,2,3,4,6-pentagalloyl glucose
EGCG	Epigallocatechin gallate
ROS	Reactive oxygen species
OCA	o-coumaric acid
SFN	Sulforaphane
PCA	p-coumaric acid
TCA	Trans-cinnamic acid
Xcc	*Xanthomonas campestris* pv. campestris
BDSF	cis-2-dodecenoic acid
